# A new era for the international representativeness of CLINICS Journal

**DOI:** 10.1016/j.clinsp.2022.100030

**Published:** 2022-04-06

**Authors:** Luiz Felipe P. Moreira, José Maria Soares Jr.

**Affiliations:** a Editor-in-Chief – CLINICS; b Hospital das Clínicas da Faculdade de Medicina da Universidade de São Paulo, São Paulo, SP, Brazil; c Associate Editor - CLINICS

Since the first *CLINICS* issue published in 2005 as a renamed journal, celebrating 60 years of uninterrupted publications of the Official Scientific Journal of the Hospital das Clínicas da Faculdade de Medicina da Universidade de Sao Paulo, in Brazil, the editorial goal was to turn it into an internationally impacting publication covering all the different medical and biomedical sciences.^1^ The mission of this open-access electronic journal continued to include the publication of quality science that originated mainly in Brazil but also changed in order to include important scientific contributions from anywhere in the world.

Over these 17 years of renamed existence, approximately 2500 original contributions and revision articles coming from more than 50 countries in all the medical specialties were published. Indexed in all the most important international databases, *CLINICS* has confirmed the degree of scientific relevance and the range of the national and international scientific research published in the journal by the improvement of different bibliometric parameters. The current CiteScore of *CLINICS* released by Scopus in 2021 was 2.6, positioning the journal between the 30% top journals in the category of General Medicine, while its Impact Factor published by Clarivate Analytics in the same year was 2.36, ranking it in the 86th position out of 169 journals in Medicine, General & Internal group.

The consolidation of this important achievement, a result of the remarkable work developed over the years by the editors who preceded us, continues to represent, on the other hand, a great challenge. Nowadays, the *CLINICS* Editorial Board has members from 13 different countries and regions committed to publishing electronically peer-reviewed research of interest to clinicians and researchers in continuous flow. Nevertheless, to face the great demand of the manuscripts submitted to publishing, which nowadays add more than 700 articles per year, the CLINICS journal improved its electronic submission system and the review and editing processes through its recent partnership with the Elsevier Publishing Company, ensuring faster responses to the authors and better editorial quality publishing of the articles selected.

The future expectations of the CLINICS journal publication under Elsevier include especially the continuing growth of its internationalizing process, increasing the participation of Section Editors, reviewers, and foreign authors. We congratulate all collaborators of *CLINICS* for the important work developed so far, and we hope to count on your continuing support to carry on this new period that has just been initiated.
